# Management of Acute Coronary Syndrome in Cancer Patients: It’s High Time We Dealt with It

**DOI:** 10.3390/jcm11071792

**Published:** 2022-03-24

**Authors:** Fabiana Lucà, Iris Parrini, Maurizio Giuseppe Abrignani, Carmelo Massimiliano Rao, Laura Piccioni, Stefania Angela Di Fusco, Roberto Ceravolo, Irma Bisceglia, Carmine Riccio, Sandro Gelsomino, Furio Colivicchi, Michele Massimo Gulizia, on behalf of Management and Quality Working Group ANMCO

**Affiliations:** 1 Cardiology Department, Grande Ospedale Metropolitano, AO Bianchi Melacrino Morelli, 89129 Reggio Calabria, Italy; massimo.rao@libero.it; 2 Cardiology Department, Ospedale Mauriziano Umberto I, 10128 Torino, Italy; irisparrini@libero.it; 3 Cardiology Department, P.O. S. Antonio Abate, 91100 Trapani, Italy; maur.abri60@gmail.com; 4 Cardiology Department, Ospedale “G. Mazzini”, 64100 Teramo, Italy; laura.piccioni@aslteramo.it; 5 Clinical and Rehabilitation Cardiology Department, Presidio Ospedaliero San Filippo Neri, ASL Roma 1, 10128 Roma, Italy; doctstefania@hotmail.com (S.A.D.F.); furio.colivicchi@gmail.com (F.C.); 6 Cardiology Department, Ospedale Lamezia Terme, 88046 Catanzaro, Italy; roberto_ceravolo@yahoo.it; 7 Integrated Cardiology Services, Cardio-Thoracic-Vascular Department, Azienda Ospedaliera San Camillo Forlanini, 00152 Roma, Italy; irmabisceglia@gmail.com; 8 Cardiovascular Department, A.O.R.N. Sant’Anna e San Sebastiano, 81100 Caserta, Italy; carmine.riccio@tin.it; 9 Cardiothoracic Department, Maastricht University, 6221 Maastricht, The Netherlands; sandro.gelsomino@maastrichtuniversity.nl; 10 Cardiology Department, Azienda di Rilievo Nazionale e Alta Specializzazione “Garibaldi”, 95126 Catania, Italy; michele.gulizia60@gmail.com; 11 Fondazione per il Tuo Cuore-Heart Care Foundation, 50121 Firenze, Italy

**Keywords:** acute coronary syndromes, atherosclerosis, cancer, cardiotoxicity, thrombosis

## Abstract

Cancer patients have an increased risk of cardiovascular disease and, notably, a significant prevalence of acute coronary syndrome (ACS). It has been shown that an elevated presence of cardiovascular risk factors in this setting leads to an interaction between these two conditions, influencing their therapeutic strategies and contributing to higher mortality. Nonetheless, cancer patients have generally not been evaluated in ACS trials, so that the treatment in these cases is still not fully known. We reviewed the current literature and discussed the best management for these very high-risk patients. The treatment strategy must be tailored based on the cancer type and stage, balancing thrombotic and bleeding risks. When the prognosis is longer than six months, especially if a clinical instability coexists, patients with ACS and cancer should be referred for percutaneous coronary intervention (PCI) as soon as possible. Moreover, an invasive strategy should be preferred in STEMI patients as well as in NSTEMI patients who are considered as high risk. On the contrary, in clinically stable NSTEMI patients, a conservative non-invasive strategy could be adopted, especially in cases of a poor life expectancy and/or of high risk of bleeding. Drug-Eluting-Stents (DES) should be the first choice if an invasive strategy is adopted. Conservative therapy could instead be considered in cancer patients with more stable CAD at an increased risk of major bleeding complications. However, the duration of dual antiplatelet therapy (DAPT) with aspirin and clopidogrel is recommended, but it should be as short as possible, whereas triple antithrombotic therapy is non-advised because it significantly increases the risk of bleeding. ACS management among cancer patients should be based on an accurate evaluation of the risk of thrombosis and bleeding. Future studies focused on choosing optimal strategies in tumor patients with ACS should be performed to treat this subset of patients better.

## 1. Introduction

Cardiovascular diseases (CVD) are the most frequent cause of late morbidity and mortality among cancer survivors [1]. The reported prevalence of cancer among acute coronary syndrome (ACS) patients ranges between 3% and 17% [2,3,4,5,6,7,8,9,10]. This association may be related to the chronic inflammation state typical of patients with neoplastic diseases [11]. Moreover, coronary lesions can also be caused by oncological therapies enhancing atherosclerosis, endothelial dysfunction, acute coronary thrombosis, and coronary spasm [12].

Remarkably, the coexistence of ACS and cancer in the same patient strongly influences prognosis [13]. The treatment in this setting is very challenging, and it should be patient-tailored [14].

Nonetheless, despite such a strong correlation between cancer and ACS, the correct management of these patients is not well defined yet.

Therefore, the aim of this study was to review the current literature on the matter and discuss the best management for these very high-risk patients.

## 2. Search Strategy

The literature search was performed in agreement with the principles of the Preferred Reporting Items for Systematic Reviews (PRISMA) statement [15].

An unrestricted literature search was performed using PubMed, Embase, and Cochrane Databases, as well as congress proceedings from major cardiology societies’ meetings. The PubMed Database was selected as the main database to perform this search.

**Figure 1:**
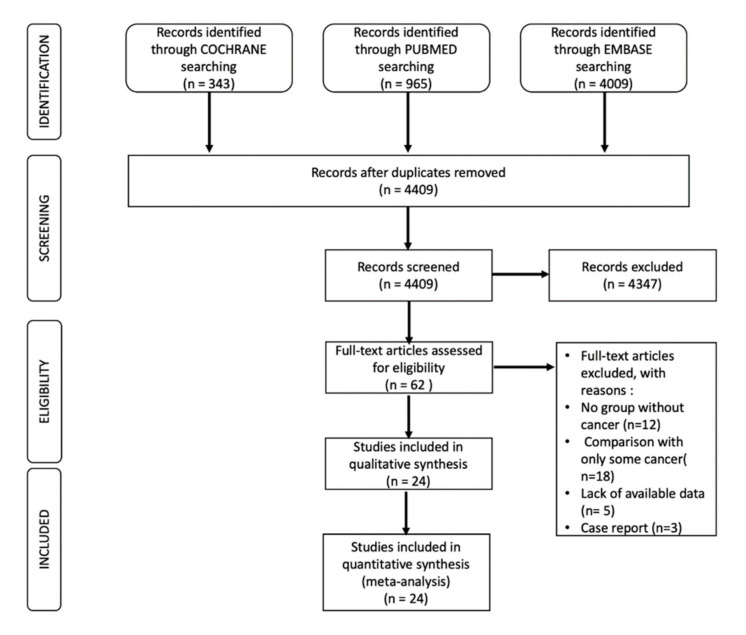
Flow Diagram Illustrating Study Selection Process.

The used PubMed search items were the following: (“Acute Coronary Syndrome” [Mesh] OR “acute coronary syndrome”) AND (“Cancer” [Mesh] OR “cancer”) AND (“Neoplasm” [Mesh] OR “neoplasm”). Articles published until January 2022 and only written in English were examined (Figure 1).

The search strategy was decided by three authors (IP, FL, MGA), and a fourth author (CR) approved the decisions.

One author (SG) performed the literature search, and the selected articles’ eligibility was assessed independently by three reviewers (IB, FC, and MMG). Corresponding authors were asked to provide full-text papers if they were not available.

From each study, information about methods, year of publication, number of patients in the treatment and control arms, duration of follow-up, age, sex, CV risk factors, medications, treatment drug, and dose were collected.

## 3. Selection Process

The article selection was based on defined inclusion criteria. These criteria were the following: (1) human studies, (2) full articles about Acute Coronary Syndrome and Cancer having a non-Cancer control population, (3) studies containing adequate information regarding the presence of Cancer and Acute Coronary Syndrome, and (4) studies including at least 10 patients.

The exclusion criteria for the article selection were: (1) non-human studies, (2) case reports, (3) previous reviews and/or meta-analyses, (4) editorials, (5) studies without data regarding both the Acute Coronary Syndrome and Cancer status of the included patients.

## 4. The Pathophysiologic Mechanism of Coronary Artery Disease in Cancer Treatment

Cancer and heart diseases share cardiovascular (CV) risk factors, such as diabetes, hypertension, obesity, smoking, and low physical activity [16]. In addition, some cancer-related conditions, such as anemia, hypoxemia, and hyperviscosity, are known to lead to ACS development because of an impaired balance between oxygen supply and consumption [17].

On the other hand, it is acknowledged that malignant hypercoagulopathy occurs in cancer patients [3]. It has been claimed that the cancer pro-coagulant factors released noticeably increase the thromboembolic risk [18]. It is well recognized that vascular wall inflammation contributes to the pathogenesis of atherosclerosis [19]. The interaction between monocytes, macrophages, and cancer cells is thought to be responsible for releasing tumor necrosis factor, interleukin-1, and interleukin-6 into the bloodstream, causing endothelial damage, which contributes to thrombosis [20]. Furthermore, it also has been well assessed that pro-coagulant and tissue factors, such as k-Ras, vascular endothelial growth factor receptor, p53, phosphatase, tensin homolog, microparticles, and exosomes, are mainly secreted in tumor patients; in addition, coagulation factors such as VII, IX, X, and XIII also play an essential role in the thrombotic process [21,22]. Moreover, it is widely accepted that mucins, containing binding sites for P- and L-selectins, are involved in leukocytes, endothelial cells, and platelets activation. Consequently, the hemostatic system’s abnormal activation and regulation in malignancy patients plays an essential role in cancer progression and cardiovascular events [23]. Finally, post-traumatic stress developed after a cancer diagnosis can be related to the increased risk of myocardial infarction [7].

Hereafter, it is well accepted that in oncologic patients, not only the classic cardiovascular risk factors, but also malignancy-related factors such as cancer type and stage and therapeutic strategies play a complex role in ACS development. In addition, considering that symptoms might be masked by other conditions such as an advanced state of the disease, several co-morbidities, the analgesic effect of treatment against cancer pain, and neurotoxic effects of chemotherapy, a silent clinical presentation commonly occurs [24].

**Figure 2:**
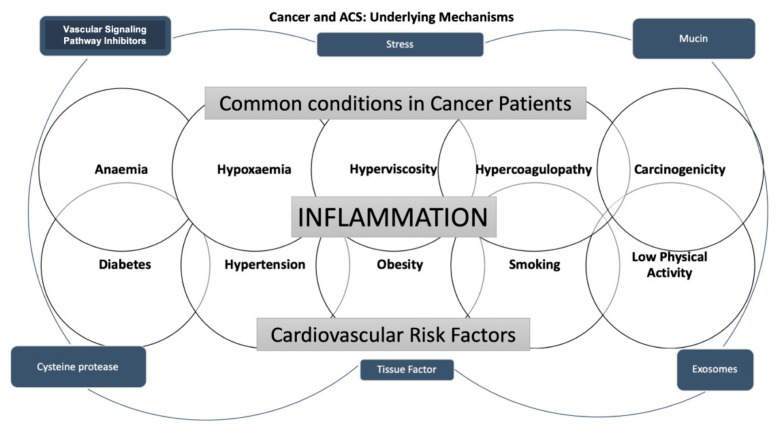
The scheme summarizes the underlying mechanisms of the associations between cancer and acute coronary syndrome.

The main mechanisms of cancer-induced CAD are shown in Figure 2.

## 5. Epidemiology

Lung, prostate, gastric, pancreatic, and breast cancer have been established to be the most frequent types associated with ACS. The highest in-hospital mortality rates and major adverse cardiovascular and cerebrovascular complications (MACCEs) have been observed in lung cancer [25]. The incidence of ACS in patients with newly diagnosed cancer is expected to be significatively higher in the first 6 months after the diagnosis and advanced cancer stages [26,27]. Indeed, its occurrence is almost two-times higher compared to the general population, with a cumulative incidence of myocardial infarction of 2.0% among cancer patients, compared to 0.7% in controls [26].

Moreover, remarkably, in clinical practice, it must be carefully taken into consideration that patients who have neoplastic diseases, such as colon cancer, also carry a significantly higher bleeding risk [26].

Finally, several concomitant conditions such as thrombocytosis, thrombocythemia, and anemia are associated with active lymphoma or leukemia patients that raise the incidence of ACS from 1.4 to 11.2% [28].

## 6. Chemotherapy and ACS Risk

Androgen suppression therapy, used to treat prostate cancer, is associated with a higher incidence of metabolic syndrome and cardiac complications. In addition, fluoropyrimidines are the second-most-frequently reported cardiotoxic agents [29], with an incidence of cardiotoxicity of 2–34% for 5-fluorouracil (5-FU) and 3–9% for capecitabine [30,31]. The most common manifestation is chest pain, which is sometimes atypical but which may be an expression of ACS [32,33].

An average time interval between the administration of 5-FU and the onset of cardiac symptoms of three days (range: 2–5 days) has been reported; this drug is frequently associated with electrocardiographic abnormalities such as ST-segment changes and T-wave inversion. The underlying mechanisms seem to be coronary vasospasm and thrombus formation; in addition, direct cellular damage to cardiomyocytes and endothelial cells is likely to occur and promotes platelet aggregation and thrombosis [34]. Moreover, cisplatin may cause arterial thrombosis through endothelial cell dysfunction, thromboxane production, and an increment of von Willebrand factor activity platelet aggregation and activation [35].

Vascular endothelial growth factor inhibitors (bevacizumab, sorafenib, and sunitinib) may induce cardiac ischemia and arterial thrombosis due to vasospasm, inflammation, and platelet activation. The mechanism appears to involve alterations in nitric oxide synthesis [36]. The incidence of angina with these drugs varies from 1 to 15%.

Another group of Tyrosine-kinase inhibitors (TI), such as nilotinib and ponatinib, may cause endothelial apoptosis with increased factor VII levels, establishing a prothrombotic state [37]. Cardiovascular events, including cerebrovascular accidents and peripheral vascular injuries, are pretty common with ponatinib [26]. Additionally, a 25% increased risk of cardiovascular events is reported in women treated with aromatase inhibitors (anastrozole and letrozole). Similarly, the use of immunomodulatory agents such as lenalidomide, pomalidomide, and the proteasome inhibitor carfilzomib has also been associated with an increase in cardiovascular events, particularly ACS [38]. Finally, the treatment with immune checkpoint inhibitors was also found to be related to an increased ACS risk, in addition to the most frequent complications, such as myocarditis. The activation of immune cells in coronary atherosclerotic plaques appears to contribute to the destabilization of atherosclerotic lesions, leading to plaque rupture and cardiovascular events [39].

The main chemotherapy agent-related mechanisms of CAD and ACS are shown in Table 1.

## 7. Radiotherapy and ACS

A direct endothelial injury can be induced by radiotherapy, since it has been well shown that a radiotherapy-related coronary artery disease (CAD) causes micro- and macrovascular progressive injuries. Involvement of the Ostia of the left main and right coronary artery is considered typical [65], with a prevalence of 85%, and it is related to the radiation field [66]. However, radiotherapy damages remain clinically silent for a long period after radiotherapy, therefore ACS is unlikely to occur during the treatment [67].

A dose of 0.50 Gy is considered the cut-off for atherosclerosis risk [68]. It has been noted that radiation produces an increase of myofibroblasts and macrophages, leading to intimal proliferation and causing a pro-thrombotic condition. Furthermore, atherosclerosis is expected to be enhanced by radiation-induced inflammation [69]. On the other hand, radiation-induced myocardial fibrosis is involved in myocardial ischemia and consequently in myocardial dysfunction, with an incremental risk of myocardial infarction proportionally increasing with the radiation duration and the age at the time of exposition [65].

In addition, even at low doses, radiations lead to microvascular damage, lowering the capillary bed’s density, reducing the vascular reserve. It has also been postulated that this damage enhances myocardial fibrosis, resulting in ischemia [65].

**Figure 3:**
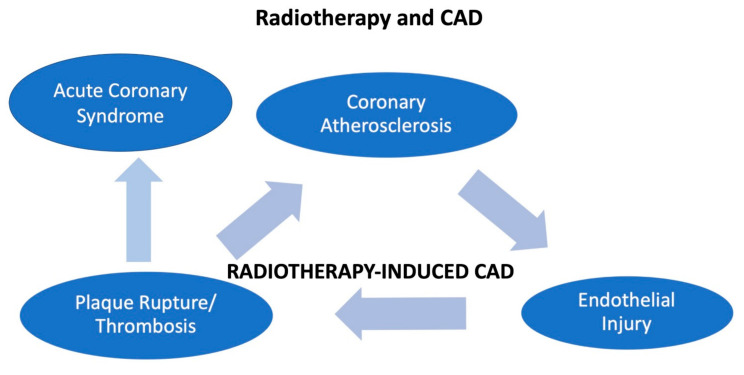
The figure shows how radiotherapy may induce coronary disease and acute coronary syndrome in patients with cancer.

The main mechanisms of radiotherapy-induced CAD are summarized in Figure 3.

## 8. Clinical Presentation

In the general population, NSTEMI is the most common clinical presentation of ACS in cancer patients [14,70].

Significantly, NSTEMI could be due not only to CAD progression, but even to concomitant conditions, including the imbalance between O_2_ supply and demand because of anemia and dehydration [17].

Indeed, myocardial infarction with nonobstructive coronary arteries (MINOCA) and Takotsubo syndrome may also occur in patients with cancer, primarily women [71].

Symptoms of ACS are generally atypical in malignancy patients, and less than one-third of them experience chest pain, and less than half have dyspnea. For this reason, careful clinical evaluation of patient history, the presence of risk factors, electrocardiogram findings, cardiac biomarkers, and echocardiographic imaging may allow ACS diagnosis in neoplastic patients [72].

A further point to consider is that these patients receive fewer cardiovascular therapies and less frequently undergo invasive strategies [2]. For patients with NSTEMI, evidence indicating a clear advantage of percutaneous revascularization treatment is scarce, especially in conditions of clinical stability, and this is due to the predisposition to bleeding, as reported by current guidelines [5,73,74].

## 9. Management

There is no complete agreement on the most appropriate treatment of cancer patients due to a lack of data to guide clinicians towards the best-tailored treatment for these patients [13].

Medical versus interventional management in cancer patients with NSTEMI should be chosen after carefully evaluating the risk/benefit ratio and the stability of the clinical status under medical treatment [5,74]. On the other hand, the approach to STEMI patients is like that of the general population [75].

In patients with ACS, a multidisciplinary team should be in charge of choosing the best treatment between conservative or invasive strategies. Patients with cancer and ACS must be immediately admitted to intensive care and monitored.

### 9.1. Cancer and Percutaneous Coronary Intervention

Percutaneous coronary intervention (PCI) improves the survival rate of ACS patients, lowering early and late cardiac events. A U.S. study analyzed all individuals undergoing PCI between 2004 and 2014 in the Nationwide Inpatient Sample: 6,571,034 PCI procedures were included, and current and previous cancer rates were 1.8% and 5.8%, respectively [76].

In the same registry, 18,052 patients had a diagnosis of lymphoma (0.25%) [77], and 15,789 patients had a diagnosis of leukemia (0.24%) [78].

In the real world, however, only a low percentage of patients with cancer and ACS perform PCI (from 54.2% for lung cancer to 70.6% for hematologic malignancies, vs. 82.3% for no cancer) [79]. In the acute myocardial infarction in Switzerland (AMIS Plus) registry, cancer patients underwent PCI less frequently (OR 0.76; 95% CI 0.67–0.88) compared to no-cancer patients [2]. Likely, patients with leukemia were less likely to undergo coronary angiography (48.5% vs. 64.5%) and PCI (28.2% vs. 42.9%) compared with those without leukemia [80]. In a retrospective analysis from a non-academic center, coronary angiography was performed in only 47% of patients with cancer, while 53% were treated conservatively [81]. Besides, in a single-center study, the median time to PCI was 10 h among the cancer patients and 7.5 h among the control group [82].

Nevertheless, some studies support the implementation of invasive treatment, irrespective of cancer stage, and show that invasive management has a better outcome. For instance, in a recent study, STEMI patients undergoing invasive treatment had a better prognosis than those medically treated when coronary angiography was performed within 72 h [83]. Moreover, a large retrospective propensity-score matching analysis showed that the few patients with neoplastic diseases treated with PCI had lower mortality, and they were less likely to have adverse cardiac events [79]. In another multicenter case-control study using a machine learning–augmented propensity score-adjusted multivariable analysis, PCI significantly reduced mortality specifically for cancer patients vs. medical management (OR 0.82, 95% CI 0.75–0.89; *p* < 0.001) [84]. In a study by Zamorano and coworkers, PCI use was associated with a better one-year survival (67% vs. 24%) [85]. Invasive treatment enhanced survival also in the presence of metastases (HR 0.37, CI 0.15–0.92) [81]. These findings are in contrast with a 10-year observational study, showing that patients with metastatic cancer had a better outcome with medical therapy compared to those treated with PCI in terms of intra-hospital mortality [70].

The Society for Cardiovascular Angiography and Interventions expert consensus statement recommends, in cancer patients, radial access to reduce the risk of vascular complications, bleeding, and MACE. In contrast, the femoral approach should be reserved for complex coronary interventions, rotational atherectomy, or the need for intra-aortic balloon pumps [86].

Regarding the devices employed, bare-metal stents have been used in the past, while now, third-generation drug-eluting stents are indicated for the lower risk of thrombosis and shorter duration of dual antiplatelet therapy (DAPT) [87]. In addition, the use of a fractional flow reserve is advised to better assess the severity of coronary stenosis. In contrast, intravascular ultrasound and optical coherence tomography can be used to ensure optimal stent apposition and expansion [86]. Current guidelines do not clearly state that invasive revascularization is not indicated in cancer. However, the European Society of Cardiology guidelines on NSTEMI suggest that an invasive strategy should be withheld in a subgroup with nonobstructive CAD and comorbidities such as cancer [74].

Cancer, as previously reported, is characterized by a hypercoagulable state, which represents one of the strongest independent predictors of stent thrombosis [88,89]. In the Kuma study, the malignant group had a significantly higher probability of target lesion revascularization at one year than the non-malignant group (*p* = 0.002); moreover, proportional hazards analyses identified malignancy as an independent predictor of target lesion revascularization (HR 2.28, 95% CI 1.3–4.0; *p* = 0.004) [90]. In the Coronary REvascularization Demonstrating Outcome Study in Kyoto (CREDO-Kyoto) PCI/coronary artery bypass grafting Registry Cohort-2, the cancer group had a trend toward a higher adjusted risk for definite or probable stent thrombosis as compared with the noncancer group (HR 1.49, 95% CI 0.99–2.16; *p* = 0.055) [91]. On the other hand, thrombocythemia, often related to chemotherapy; anemia, due to blood loss, malnutrition, and infection; and hepatic dysfunction, associated with coagulation disorders, increase hemorrhagic risk [92,93].

Besides, patients with cancer had more comorbidities and were older.

Using a nationwide quality registry of all patients admitted for a first MI in Sweden, cancer was associated with major bleeding during a median follow-up of 4.3 years (HR 1.45; 95% CI 1.34–1.57) [94].

Bleeding is related to the type and stage of cancer; the gastrointestinal tract tumors are at a greater risk [95]. Kwok et al., in the US Nationwide Readmission Database, observed a higher 90-day readmission for bleeding after PCI in patients with active cancer (4.2% in colon, 1.5% in lung, 1.4% in prostate, 0.6% in breast, and 1.6% in all cancers) compared to 0.6% among patients with no cancer [96]. In a U.S registry on PCI interventions in cancer patients, colon and prostate cancer were associated with bleeding risk (respectively, OR 3.65, 95% CI 3.07–4.35 and OR 1.41, 95% CI 1.20–1.65) [76]. In the same registry, the diagnosis of Hodgkin Lymphoma was associated with increased odds of bleeding complications (OR 1.12, 95% CI 1.05 to 1.20), whereas these odds were not significantly associated with a non-Hodgkin’s diagnosis [77].

Furthermore, a leukemia diagnosis was associated with significantly increased odds of in-hospital bleeding (OR: 1.87; 95% CI: 1.56–2.09) [78].

After PCI, the active cancer group had clinically relevant bleeding during both DAPT and single antiplatelet therapy (SAPT) periods, and a multivariate Cox regression hazard analysis revealed cancer activity as a significant independent risk factor for bleeding (*p* = 0.023) [97].

A PCI registry analysis performed in Berna, including 13,000 patients, of which 10% had a historical diagnosis of cancer, found no difference in major ischemic events but an increased risk of major bleeding at one year [87].

In the BleeMACS project, a multicenter observational registry enrolling patients with ACS undergoing PCI worldwide in 15 hospitals, after one year, patients with cancer more often experienced bleedings (6.5% vs. 3%, *p* < 0.001), and in a multiple regression analysis, the presence of cancer was the strongest independent predictor for bleedings (HR 1.5, CI 1.1–2.1, *p* = 0.015) [6]. According to ESC NSTEMI guidelines, active malignancy (excluding non-melanoma skin cancer) within the past 12 months is a major bleeding risk factor [74].

### 9.2. Pharmacological Treatment of ACS in Cancer Patients

In cancer patients with ACS, there is an increased risk of both thrombotic and hemorrhagic events [93]. Therefore, the clinical decision on antiplatelet drugs and DAPT, aimed at mitigating the risk of stent thrombosis, is challenging for cardiologists and oncologists.

Observational data show that cancer patients with ACS were less likely to receive guideline-recommended drugs [98].

Yusuf et al. retrospectively analyzed 456 malignancy patients with MI, including 70 cases of STEMI, and, among these patients, only 211 (46.3%) received aspirin. However, in a retrospective study from the University of Texas, the one-year survival was higher in patients treated with aspirin (34%) than in those without (18%), and aspirin use was associated with a 23% decreased risk of death (HR: 0.77, 95% CI: 0.60–0.98, *p* = 0.033) [99]. Furthermore, the use of aspirin was a significant predictor of improved survival also when adjusted for the presence of metastases (HR 0.39, CI 0.16–0.94) [81].

A particular set of patients with cancer and ACS have thrombocytopenia. In a study on this population, subjects who did not receive ASA had a seven-day survival rate of 6% compared with 90% in those who had ASA (*p* < 0.0001) without severe bleeding complications [100].

In another retrospective study of cancer patients with chronic thrombocytopenia who underwent cardiac catheterization for ACS, aspirin therapy (alone or in combination with clopidogrel) was used in 66 patients (67.3%), whereas 27 patients (27.6%) were on dual antiplatelet therapy with a low incidence of bleeding complications and neither procedure-related antiplatelet nor therapy-related cerebrovascular events [101]. A single-center prospective study in cancer patients with a recently placed (1–12 months) Drug-Eluting System (DES) demonstrated that Optical Coherence Tomography (OCT) imaging allows the identifying of low-risk cancer patients who may safely discontinue DAPT and proceed with cancer-related surgery or procedures [101]. Finally, in the AMIS Plus registry, cancer patients received P2Y12 blockers (OR 0.82; 95% CI 0.71–0.94) and statins (OR 0.87; 95% CI 0.76–0.99) less frequently [2].

### 9.3. Platelet Concentration Thresholds for Individual Elements of the Therapy

The current expert consensus recommendation by the Society for Cardiovascular Angiography and Interventions (SCAI) sets the lower level of platelet count for aspirin therapy at 10,000/µL and for DAPT (aspirin and clopidogrel) at 30,000/µL [86]. In the catheterization laboratory, a reduced bolus of unfractionated heparin of 30–50 U/Kg is required in patients with platelets <50,000 µL [86]. Platelet transfusions may be considered in thrombocytopenic patients who develop bleeding during or after cardiac catheterization or when there is a rapid drop in platelets or coagulation abnormalities or if the platelet count falls below 10,000/µL, whereas prophylactic platelet transfusion is not recommended unless required by the oncology/hematology team [5,13,86].

If the platelet count falls below 30,000/µL, revascularization and DAPT should be decided after a preliminary multidisciplinary evaluation (interventional cardiology/oncology/hematology) and a risk/benefit analysis [86]. Ticagrelor, prasugrel, and IIB-IIIA inhibitors should be avoided unless platelet counts are more than 50,000/mL [13,86].

## 10. Discussion

Cancer and CVD are the leading worldwide causes of mortality [102]. 

The causal relationship between these two entities is partially due to the fact that they share several modifiable and non-modifiable risk factors [16].

Moreover, cancer survival should be considered an independent CV risk factor [103]. A proinflammatory and hypercoagulable condition with increased platelet activation and aggregability typically occurs in cancer, increasing the prevalence of ACS [72,104].

Moreover, while, on the one hand, novel cancer treatments had a significantly improved cancer survival, on the other hand, this has led, at the same time, to a rise in CVD incidence [105].

A new diagnosis of coronary heart disease (CAD) is more frequent by six months following the diagnosis of cancer, confirming this link [106]

Therefore, the occurrence in the same patient of cancer and CAD requiring PCI is significantly rising [76].

However, remarkably, cancer has not been included in ischemic and bleeding scores such as GRACE [107] and CRUSADE [108], despite this relevant clinical influence [109,110,111,112]. Furthermore, another point worth mentioning is that patients with concomitant cancer and CAD have not been included or have been poorly represented in most ACS trials [113].

Consequently, the optimal strategy in cancer patients is still a dilemma, and PCI efficacy and safety are largely debated, despite the high prevalence of ACS in this subset of patients [26]. However, the following aspects related to clinical properties should be carefully recognized:

(1) The differentiation between active cancer from the history of cancer; (2) the type and stage of cancer; (3) the presence of metastases [5].

Cancer, particularly in its active form, is an independent predictor of ischemic and bleeding complications after PCI. Indeed, thrombocytopenia and anemia, which have been well-recognized as predictors of bleeding, occur in 10–25% and 30–90% of cancer patients, respectively [113].

In-hospital mortality, MACCEs, stroke, and bleeding have been observed significantly higher in acute myocardial infarction (AMI) patients with current cancer than in those whose cancer was previously diagnosed [25].

A higher rate of 90-day readmission either for AMI or for bleeding has been reported by Kwok et al. in 183,268 current cancer patients (9.5% of 933,324 patients who had undergone PCI), compared to no cancer patients [96].

Moreover, less frequent use of PCI in patients with active cancer has been demonstrated by Bharadwaj et al. [25]

Regarding the subtype of neoplasm, lung cancer and colon cancer have been associated with a worse outcome [25].

Lung cancer is strongly associated with a more significant increase in in-hospital mortality.

Conversely, a significatively higher risk of bleeding but not of mortality has been described in current colon cancer [76].

Accordingly, in the study conducted by Kwok, the AMI readmission occurred more frequently in patients with active lung cancer (12.1%) and colon cancer (10.8%) [96]. In contrast, active colon cancer was closely associated with bleeding readmission (4.2%) [96].

However, the lung cancer subgroup is the most likely to receive no treatment, with only 21% of the PCI performed compared to 43.8% of patients without cancer [25].

Nonetheless, no increase in in-hospital mortality in prostate and breast cancer has been observed [25].

Furthermore, a worse outcome has been reported in patients with ACS and acute myeloid leukemia (AML) [80].

This subset of patients is less likely to receive PCI; however, when an invasive strategy is adopted, a significantly higher in-hospital mortality and bleeding rate have been observed [80].

Contrary to non-Hodgkin’s lymphoma [77], a higher risk of in-hospital death, vascular complications, and bleeding have also been shown in Hodgkin’s lymphoma [78].

However, not only the type of cancer, but also the metastases status has been demonstrated to influence the outcome deeply [25].

A greater risk of mortality, bleeding, and stroke has been described in metastatic patients compared to other patients [25].

Independently of the type of cancer, metastasis occurrence has been well recognized as a factor of worse in-hospital outcomes [105]. Nonetheless, in breast and colon cancers, stroke incidence has not been influenced by metastases status [25].

The analysis of 49,515 metastatic cancer patients affected by ACS showed a beneficial effect of PCI in terms of mortality compared to conservative medical therapy in this subset [70].

Therefore, except for metastatic patients [70], adopting an invasive strategy seems to be related to a better outcome than differing procedures [99,114,115], despite the increased risk of periprocedural complications [90,116].

However, for the risk of surgical procedures or chemotherapies or bleeding, cancer patients with ACS are more likely to receive any invasive strategy [117].

Data from the US National Inpatient Sample (NIS) on 6,563,255 patients referred for AMI between 2004 and 2014 (186,604 with active cancer and 409,697 with a history of cancer) showed that conservative management (medical treatment without PCI) had been largely preferred in the cancer patients [25].

These findings have been supported by the propensity score-matched analysis on 38,932 active cancer patients (1,870,815 referred for STEMI), which confirmed that an invasive strategy was less likely to be performed in cancer patients, even though a strong association between PCI and in-hospital all-cause mortality and MACCEs lowering has been shown in this cohort [79].

In light of the present situation, the underuse of PCI in this high-risk patient category is one of the most relevant reasons for the lower survival rate [91].

A one-year survival of 26% has been reported by Yusuf in a retrospective analysis on 456 cancer patients with AMI (386 NSTEMI and 70 STEMI), showing that only 2.8% and 5.7% of NSTEMI and STEMI patients, respectively, underwent revascularization [99].

Undoubtedly, one of the most relevant concerns which strongly limits the invasive strategy in cancer patients [106] is the perspective of dual antiplatelet therapy (DAPT) required after PCI [117,118]. 

DAPT with aspirin (300/75–100 mg) and clopidogrel (300–600/75 mg) is recommended in this subset [72], whereas newer P2Y12 antagonists such as ticagrelor and prasugrel should be avoided considering the high bleeding risk and lacking data on effectiveness and safety in cancer patients [5]. Besides this, the duration of DAPT should be as short as possible (1–3 months). Aspirin and clopidogrel use is allowed if the platelet count is >10,000/μL and >30,000/μL, respectively [72].

Furthermore, aspirin and clopidogrel should be preferred in patients whose cancer diagnosis is shorter than 12 months and if other bleeding risk factors occur [5].

However, several scores (PRECISE-DAPT, PARIS) used to assess the risk and/or benefit of short vs long-term antiplatelet therapy after PCI have not been validated in patients with cancer [119]. The Academic Research Consortium for high bleeding risk (ARCHBR) criteria has been suggested for assessing the bleeding risk in this setting of patients [120].

Using a proton pump inhibitor and not associating with non-steroidal anti-inflammatory drugs should also be kept in consideration. 

It also has been well assessed that in cancer patients, atrial fibrillation (AF) and CAD often coexist [119]. These two entities are often associated [121], and 10% of patients undergoing PCI for CAD have AF [122].

Moreover, other comorbidities such as valvular heart disease (VHD) with a mechanical prosthesis or Venous Thromboembolism (VTE) frequently occur in cancer patients [123].

However, the concomitant use of OAC with DAPT, known as triple antithrombotic therapy (TAT) [124], which would be required in the absence of cancer [125], is non-advised in cancer patients [3] because of the significantly higher risk of bleeding [122,126,127].

Furthermore, in this cohort, OAC should be associated with clopidogrel [5]. The single antiplatelet agent use should be as short as possible [5].

Thus, clinical decision-making in cancer patients who need oral anticoagulation therapy (OAC) for one of the above-mentioned reasons and who are experiencing an ACS is particularly complex because of the lack of evidence-based management [124,128,129,130].

Moreover, cancer has not been included in the most common risk scores such as CHA2DS2-VASc [131] and HAS-BLED [132] or ABC [133].

Therefore, balancing the higher ischemic and bleeding risk [88,89] is one of the most challenging features in this subset.

Finally, a radial approach and the use of coronary imaging such as intravascular ultrasound (IVUS) and optical coherence tomography (OCT) have also been proposed as suitable strategies for lowering the bleeding risk in cancer patients [5].

In patients undergoing invasive procedures, Drug-Eluting-Stents (DES) should be the first choice if a PCI would be performed [5]. In these settings, newer DES, such as BioFreedom stent [134], Synergy [135], and Nienke platforms [136], or polymer-based zotarolimus-eluting stents [137] are safe and effective for an abbreviation of DAPT.

## 11. Limitations

This systematic review has some limitations that have to be pointed out.

Firstly, the search strategy results were limited to reports published in the English language, which may lead to language bias, as there is a risk that potentially relevant studies reporting the impact of cancer on patients with ACS, particularly in low-income and middle-income countries, were not included and remain unknown. Secondly, the current literature search was limited to three large multidisciplinary databases (PubMed, Cochrane, and Embase).

Third, we also did not include “acute myocardial infarction” or “unstable angina” as search terms. Therefore, it is possible that some studies not reporting the term “acute coronary syndromes” were missed. These limitations may affect the comprehensiveness of the search. However, we tried to overcome these limitations by checking the reference lists of included articles to identify additional studies.

Regarding study quality, we observed wide heterogeneity in all domains within the selected trials, including sample size, baseline characteristics, geographical variations, study design, methods, eligibility criteria, different medications, adjustment for confounders, and analyses. Besides, long-term follow-up data were lacking from most trials, as they limited their outcomes to in-hospital, 30-day, and one-year all-cause mortality; it would be of great interest to investigate prognosis over the life course rather than using short time horizons.

Furthermore, it is also worth mentioning that a quantitative meta-analysis of included studies could not be undertaken due to their methodological flaws and vast heterogeneity. This issue will only be resolved by future research, more carefully documenting and reporting optimal strategies of management of ACS in cancer patients.

## 12. Conclusions

ACS in cancer patients has become an increasingly common challenge in clinical practice.

Considering the little evidence from literature data, ACS management among cancer patients should be based on evaluating the risk of thrombosis and bleeding.

Treatment should be tailored to each patient, not only according to the ACS sub-type (unstable angina, NSTEMI, and STEMI), but also considering the stage and type of cancer, anemia and thrombocytopenia, bleeding risk, hemodynamic stability, life expectancy, previous or ongoing cancer therapies, future treatment plans, planned surgery, and prognosis.

When the prognosis is longer than six months, especially if a clinical instability coexists, patients with ACS and cancer should be referred for PCI as soon as possible. Moreover, an invasive strategy should be preferred in STEMI patients as well as in NSTEMI patients who are considered at high-risk. On the contrary, in clinically stable NSTEMI patients, a conservative non-invasive strategy could be adopted, especially in the case of a poor life expectancy and/or of a high risk of bleeding, such as those with metastases, coagulopathies, or thrombocytopenia.

Regarding antithrombotic treatment, a DAPT with aspirin and clopidogrel instead of newer P2Y12 antagonists is recommended, and, besides, its duration should be as short as possible, whereas TAT therapy is not advised.

A multidisciplinary approach is required to treat cancer patients presenting with acute CV complications. Antineoplastic therapy should also be discussed within the multidisciplinary team with an accurate evaluation of its implication on ACS development.

Future studies focused on choosing optimal strategies in tumor patients with ACS should be performed to treat this subset of patients better.

## Figures and Tables

**Table 1:** Mechanism of Antineoplastic Drug-Induced Cardiotoxicity.

Class	Agents	Cardiotoxic Effects
Immunomodulatory	Lenalidomide [40] Pomalidomide [41] Immune Checkpoint Inhibitors [42,43]	Endothelial Dysfunction → Destabilization of atherosclerotic lesions → Plaque rupture → Cardiovascular events
Anti-microtubule	Paclitaxel [44]	Vasoconstriction, Endothelial injury → Cardiovascular events
Proteasome Inhibitor	Carfilzomib [45] Bortezomib [46]	Cardiac ubiquitin–proteasome dysfunction → Endothelial injury→ Cardiovascular events
Aromatase Inhibitors	Anastrozole [47] Letrozole [47]	Vasoconstriction, Endothelial injury → Cardiovascular events
Anti-metabolites	5-fluorouracil (5-FU) [30] Capecitabine [48] Gemcitabine [49] Nilotinib [50]	Coronary Vasospasm Thrombus Formation Direct cardiomyocytes and endothelial cells damage → Increase of Von Willebrand Factor’s activity → Cardiovascular events
BRC-ABL tyrosine kinase inhibitors	Nilotinib [50] Ponatinib [51]	Coronary Atherosclerosis Endothelial Apoptosis → Increase of Factor VII Levels → Prothrombrotic state → Cardiovascular events (cardiac, cerebrovascular, and peripheral events)
Vascular Endothelial Growth Factor Inhibitors	Bevacizumab [52] Sorafenib [53] Sunitinib [54] Pazopanib [55] Regorafenib [56] Axitinib [57] Ramucirumab [58] Aflibercept [59]	Vasospasm Inflammation Platelet Activation → Cardiac ischemia and arterial thrombosis
Alkilating agents	Cyclophosphamide [60,61]	Endothelial dysfunction → platelet aggregation and activation → cardiovascular events
Vinca-alkaloids	Vincristine [62]	Thrombus Formation Endothelial Injury → Cardiovascular Events
Platinum	Cisplatin [63]	Endothelial dysfunction → Thromboxane Production → Thrombus Formation → Platelet aggregation and activation → Cardiovascular Events
Anti-tumor antibiotics	Bleomycin [64]	Endothelial dysfunction → Platelet aggregation and activation → Cardiovascular Events
